# Salicylic Acid, a Plant Defense Hormone, Is Specifically Secreted by a Molluscan Herbivore

**DOI:** 10.1371/journal.pone.0086500

**Published:** 2014-01-22

**Authors:** Julia Kästner, Dietrich von Knorre, Himanshu Himanshu, Matthias Erb, Ian T. Baldwin, Stefan Meldau

**Affiliations:** 1 Department of Molecular Ecology, Max-Planck-Institute for Chemical Ecology, Jena, Germany; 2 Phyletisches Museum, Friedrich-Schiller-Universität Jena, Jena, Germany; 3 Root-Herbivore Interactions Group, Max-Planck-Institute for Chemical Ecology, Jena, Germany; University of Dundee, United Kingdom

## Abstract

Slugs and snails are important herbivores in many ecosystems. They differ from other herbivores by their characteristic mucus trail. As the mucus is secreted at the interface between the plants and the herbivores, its chemical composition may play an essential role in plant responses to slug and snail attack. Based on our current knowledge about host-manipulation strategies employed by pathogens and insects, we hypothesized that mollusks may excrete phytohormone-like substances into their mucus. We therefore screened locomotion mucus from thirteen molluscan herbivores for the presence of the plant defense hormones jasmonic acid (JA), salicylic acid (SA) and abscisic acid (ABA). We found that the locomotion mucus of one slug, *Deroceras reticulatum,* contained significant amounts of SA, a plant hormone that is known to induce resistance to pathogens and to suppress plant immunity against herbivores. None of the other slugs and snails contained SA or any other hormone in their locomotion mucus. When the mucus of *D. reticulatum* was applied to wounded leaves of *A. thaliana*, the promotor of the SA-responsive gene *pathogenesis related 1* (PR1) was activated, demonstrating the potential of the mucus to regulate plant defenses. We discuss the potential ecological, agricultural and medical implications of this finding.

## Introduction

Plants have evolved various mechanisms to detect herbivore attack. They can respond to insect herbivores via perception of mechanical damage and chemical cues that are released from tarsal pads, insect eggs or oral secretions of insects [1]. The detection of specific herbivore-derived cues, so called herbivory-associated molecular patterns (HAMPs), allows plants to distinguish herbivore attack from wounding and often leads to the activation of herbivore-specific defense responses [2]. Perception of HAMPs generally triggers the induction of jasmonate biosynthesis, including jasmonic acid (JA) and its active isoleucine conjugate JA-Ile [2]. Activation of the JA pathway, in turn, controls the biosynthesis of defense metabolites that increase a plant’s resistance to herbivores [3].

An increasing number of studies indicate that herbivores can also release effectors that suppress jasmonate-dependent immune responses [4]. Glucose oxidase activity, present in oral secretions of lepidopteran herbivores and aphids, induces salicylic acid (SA, 2-hydroxy benzoic acid) signaling, leading to the suppression of JA-dependent defenses, which ultimately increases the performance of herbivores [5], [6]. Oviposition often precedes the onset of herbivore feeding and in some cases eggs are equipped with chemical cues that suppress jasmonate-dependent defenses in the plant. Oviposition by *Pieris brassicae* on *A. thaliana*, for example, was found to reduce the level of anti-herbivore defense of host plants by inducing SA production and expression of SA marker genes, such as the *pathogenesis related 1* (PR1) gene [7], [8].

Some herbivores, including leaf miners and gall-inducing insects, also release plant growth hormones (cytokinins and auxin) that modulate a plant’s physiology [9], [10]. However, these hormones are likely to originate from microbial symbionts [11], [12]. It remains unknown if herbivores directly release phytohormones, that may alter plant defense responses, as has been described for various pathogens [13], [14].

Until now, only insect herbivores were analyzed with regard to their defense suppression activity. Whether molluscan herbivores, which are major pests in many agricultural and ecological settings, also employ strategies to suppress plant defense responses is currently not known. We recently found that mollusks are natural herbivores of *A. thaliana* in Germany and in the United States [15]. This plant employs jasmonate biosynthesis and signaling to increase its resistance against slugs and snails [15]. In addition, *A. thaliana* responds to the slime of molluscan herbivores by activating the JA pathway and increasing its resistance to subsequent herbivory [15], [16]. Here, we examined the hypothesis that slugs and snails supplement their locomotion mucus with plant hormones to alter plant defense responses.

## Materials and Methods

### Mollusk Cultivation

The snails and slugs used for experiments included species that occur sympatrically with *A. thaliana* (*Arion fucus*, *Deroceras laeve*, *Deroceras reticulatum*, *Helix pomatia*, *Lehmannia marginata*, *Malacolimax tenellus*, *Helicella itala*, *Perforatella incarnata*, *Trochulus hispidus*, *Monacha cartusiana*, *Succinea putris*, *Xerolenta obvia*), as well as one exotic species (*Achatina fulica*). All mollusks were cultivated in a climate chamber (Snijders scientific, Tilburg, The Netherlands), under constant humidity of 80%, a temperature of 16–20°C, and short day conditions (9.5 h light/13.5 h dark). All slugs and snails were collected around Jena (GPS: 50.92050°N, 11.61162°E) and Martinfeld (51.28634°N, 10.17949°E, Thuringia, Germany). No specific permissions or approvals were required for collecting slugs and snails at these locations and no dangered or protected species were collected. *Achatina fulica* is an important pest in many tropical countries and was provided by Dr. Gustavo Bonaventura. Different numbers of snails were separated in large plastic boxes (OKT easyfresh, Sternwede, Germany; 26.5×13×15 cm) dependent on their size. Slugs were maintained in smaller boxes (10.5×4×8 cm) and the number of individuals in one box depended on their size and social compatibility. Potatoes, lettuce and cucumber were provided, with the addition of cuttlebone for calcium (ArtNr.5050, TRIXIE Heimtierbedarf GmbH & Co. KG, Tarp, Germany). The food was changed twice a week and the boxes were cleaned and provided with fresh tissue paper and moistened with tap water.

### Plant Cultivation

*Arabidopsis thaliana* plants for experiments were grown in a standard growth substrate (Fruhstorfer Nullerde:vermiculite:sand, 8∶1:1) in a climate chamber (21°C, 55% relative humidity and 130 µmol m^−2^s^−1^ photosynthetically active radiation) with a photoperiod of 10 h light/14 h dark.

### *Deroceras Reticulatum* Behavior

Observations were made under short day conditions, 10 hours light (white light: 380–800 nm) and 14 hours dark (infrared light: 725–925 nm). *D. reticulatum* behavior was recorded using a Logitech Webcam 600 and the program Webcam XP. Four individuals were placed in a plastic box (26.5×13×15 cm) filled with soil, wood, stones and one *A. thaliana* (Col-0) plant. Every ten minutes, the behavior of *D. reticulatum* was recorded by taking a picture of the setup. Pictures were edited with Adobe Photoshop CS5 12.0 and converted into time-lapse videos with VirtualDub 1.9.11.0. File size was reduced using Mac X Free MP4 Video converter.

### Hormone Analysis

Locomotion mucus of different slugs and snails were collected by allowing them to crawl over pre-cleaned fastener (Velcro, http://www.velcro.com/Products/For-Fabrics/Sew-On/Sew-On.aspx) that was washed with water and 99% Ethanol and dried at 80°C for 5 h prior to use. To collect only freshly produced mucus, all slugs and snails were allowed to crawl over tissue paper before slime was collected. After initial screening, the analysis of *D. reticulatum* mucus was replicated four times. Phytohormones were extracted as described in [6]. In brief, the fastener (with and without mucus) was extracted with 1 mL Ethyl acetate (spiked with labeled internal standards for salicylic acid (50 ng per sample), abcisic acid (50 ng per sample), jasmonic acid (JA, 10 ng per sample) and JA-isoleucine (50 ng per sample)), vortexed for 10 min and centrifuged at 16.000 g. Supernatant was evaporated and re-dissolved in 200 µL 70% MeOH. LC-MS analysis was done as described [17]. Leaf treatments were done as described in GUS-staining experiments. Leaves were harvested at indicated time points, flash frozen in liquid nitrogen and extracted as described above, with the exception that 500 µL 70% MeOH was used for re-dissolving the dried leaf extracts.

### GUS-staining Experiments

PR1::GUS plants were provided by Philippe Reymond (Department of Plant Molecular Biology, University of Lausanne, CH-1015 Lausanne, Switzerland). Leaves of four weeks old *A. thaliana* PR1::GUS plants were wounded with a fabric pattern wheel and locomotion mucus was applied by allowing *D. reticulatum* to crawl over the wounded leaves. Wounding alone and application of water served as control. Water application was done by gently striking the leaf with one finger (with glove) to mimic slug movement. GUS staining was performed 48 h after treatments as described previously [7]. Briefly, leaves were incubated overnight at 37°C in X-Gluc solution (Sigma) and de-stained twice in 99% Ethanol, followed by incubation in chloral hydrate solution (80 g chloral hydrate, 10 ml glycerol, 30 ml water) until leaves were completely transparent. Pictures were taken with a Canon Powershot SD1000 camera (www.canon.de).

## Results

**Figure 1:**
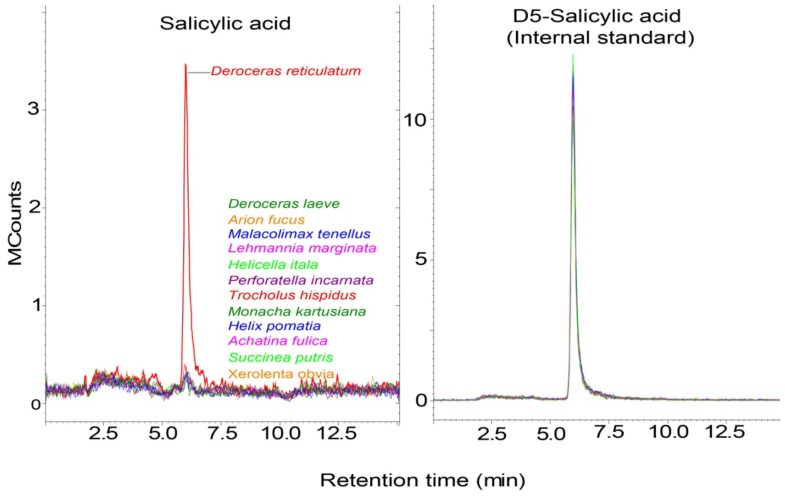
*Dercoreas reticulatum* contains salicylic acids in its locomotion mucus. LC-MS spectra of salicylic acid (left) and its internal standard (right) from locomotion mucus extracts from 13 different molluscan herbivores.

**Figure 2:**
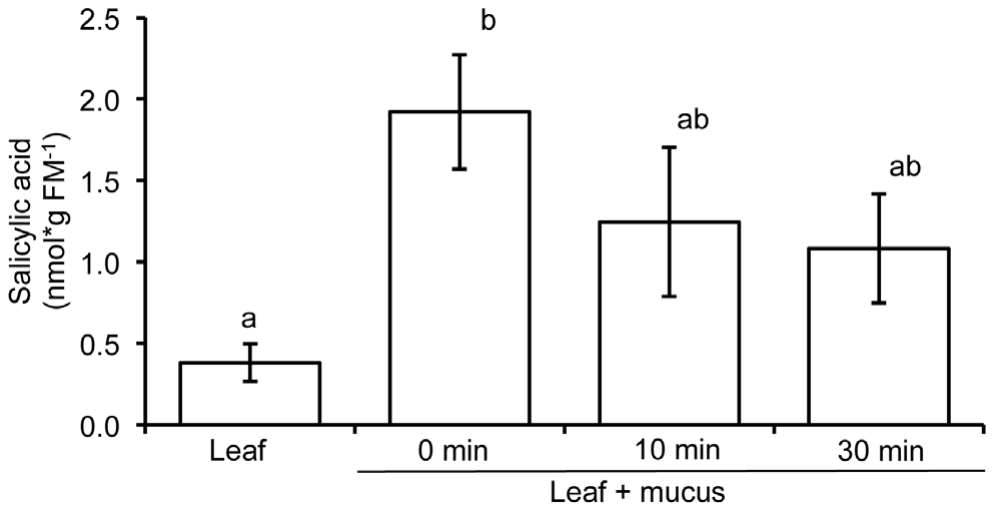
Increased salicylic acid levels in extracts of *Arabidopsis thaliana* leaves supplied with locomotion mucus of *Deroceras reticulatum*. Average ± SE of five biological replicates. Different letters indicate significant differences between treatments for each time point (ANOVA, Turkey HSD, P<0.05).

Slugs and snails secrete mucus, which aids their locomotion and protects them from desiccation. These characteristic “slime” trails persist on plants long after the slugs and snails are gone (Video S1, [15], [18]). *A. thaliana* responds to mucus of molluscan herbivores [15], [16]. Here we tested if locomotion mucus of slugs and snails contains phytohormones that may alter anti-herbivore plant defenses, including SA, JA, JA-Ile and ABA [2]. We extracted locomotion mucus from 13 different slug and snail species that occur sympatrically with *A. thaliana* and analyzed their phytohormone levels. Although we did not find any traces of JA, JA-Ile and ABA, the mucus of one slug species (*D. reticulatum)* contained significant amounts of SA (Figure 1). Since all slugs and snails, which were cultivated under similar conditions and were cleaned before extraction (see material and methods) we can exclude that SA was carried over from the cultivation boxes. The extraction of *D. reticulatum* locomotion mucus was repeated 4 times and we always found the characteristic molecular ion of SA, although the concentration of SA varied between 2.8–15 nmol*g mucus fresh mass^−1^. We also treated leaves with locomotion mucus of *D. reticulatum* and found a significant increase in SA levels from this leaf material, when compared to non-treated control leaves (Figure 2).

**Figure 3:**
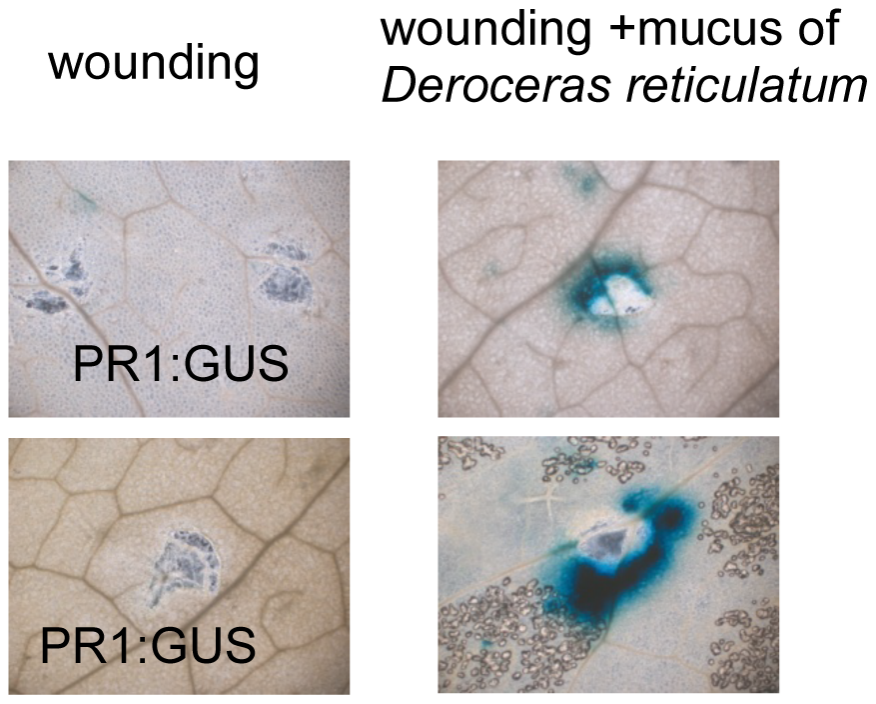
Locomotion mucus of *Deroceras reticulatum* increases *PR1* promotor activity in transgenic *Arabidopsis thaliana* PR1::GUS plants. Leaves were wounded and water or *D. reticulatum* locomotion mucus was applied to wounds. Blue color indicates *PR1* promotor activation. Pictures from two independent experiments are shown.

It was previously shown that butterfly egg depositions increase SA levels in leaves of *A. thaliana* and that this leads to higher expression of SA marker genes, including *PR1* [8]. We used transgenic *A. thaliana* plants that express β-glucuronidase (GUS) under the control of the promotor of the SA-responsive *PR1* marker gene (PR1::GUS), to test if simulated slug feeding leads to increase in *PR1* promotor activity. Applying *D. reticulatum* locomotion mucus to wounded leaves of *PR1::GUS* plants increased β-glucuronidase activity, but not after wounded leaves were treated with water (Figure 3). In our short-term experiment (48 h treatment), we found that PR1-promotor activity was only activated at wounding sites of mucus-treated leaves. Leaves supplied with mucus alone did not lead to activation of PR1::GUS activity. Wounding probably increased the absorption of SA into leaf tissue, leading to the activation of SA-responsive gene expression.

## Discussion

Grazing by mollusks can be the dominant form of herbivory in various ecosystems [19]–[21]. Selective damage by slugs and snails during the seedling stage can change plant community composition and ecosystem functioning [22]–[25]. Slugs, such as *D. reticulatum* are also serious pest in many crops [26]. Despite their importance, little is known about the chemical communication between plants and molluscan herbivores. Here, we found that the locomotion slime of *D. reticulatum* contains SA and induces the promotor of an SA-related gene in rosette stage *A. thaliana* plants. Since none of the other molluscan herbivores contain SA in their locomotion mucus, our data suggest that the excretion of SA is highly species-specific. We found no correlation between SA levels and the age of the slugs or the time that they have been in captivity (data not shown), indicating that the production is not influenced by environmental conditions.

### Origin of SA

In addition to its well-known occurrence in plant tissues, SA is widely found in the animal kingdom. Mammals contain levels of SA in their blood and in addition to its origin from plant material; there is evidence that SA can be synthesized from ingested benzoic acid [27]. Whether the SA we found in the locomotion mucus of *D. reticulatum* is sequestered from plant material or synthesized via plant-derived benzoic acid by the slugs requires further work. Supplying food of *D. reticulatum* with labeled SA or precursors of SA will help to answer these questions. Since several genera of bacteria are known to synthesize SA [28], [29], this metabolite could also be provided by microbes living in the mucus of *D. reticulatum*. Treating *D. reticulatum* with antibiotics may reveal its potential microbial origin.

### Ecological Implications

SA is an important plant hormone that regulates various aspects of plant growth and development, including regulation of photosynthesis [30]–[33] and cell expansion [34]. SA mediates plant defense against biotrophic and hemibiotrophic pathogens and some sucking insects [35]. Priming of SA-related defense responses increases disease resistance and plant fitness in the field [36]; however, activating the SA-pathway reduces plant growth and fitness in pathogen-free environments [37], [38]. These data demonstrate that SA can profoundly influence plant interactions with their environment. Whether SA provided by *D. reticulatum* to plant tissues changes plant growth or their resistance to pathogens and herbivores requires further research. When *D. reticulatum* was provided with *A. thaliana* as sole food source, the slugs supplied locomotion mucus for more than one day before they started consuming leaves (Video S1). It is tempting to speculate that the gap in feeding was caused by the time required to suppress anti-herbivore defenses via SA in the mucus. However, *A. thaliana* might also not present a suitable food source for *D. reticulatum* and our observations were done under very artificial conditions. Whether *D. reticulatum* applies mucus to other plant species prior to leaf consumption in natural settings and if this alters plant growth and defense against herbivores and pathogens requires further observations.

### Cultural Implications

The potent analgesic and antipyretic activities of plant tissue extracts, such as willow bark, in humans had been known for many centuries before the identification of SA, as the likely active compound [39]. SA is also used as medical tinctures against warts [40], [41]. Interestingly, rubbing the slime of slugs over warts has been used as anti-wart treatments as described in folklore books in the 19^th^ century [42]. The concentration of SA that we found in the locomotion mucus of *D. reticulatum* is several orders of magnitude lower than that of in commercially available wart treatments. Other slug secretions, such as the thick mucus secreted by slugs as defense during attack, may contain higher concentrations of SA, which may justify its use as wart cures. These secretions are currently being investigated.

## Supporting Information

Video S1 ***Deroceras reticulatum*** **activity on** ***Arabidopsis thaliana*** **.**
(M4V)
